# Auditory Middle Latency Responses: a study of healthy children

**DOI:** 10.1016/S1808-8694(15)30085-9

**Published:** 2015-10-19

**Authors:** Ana Claudia Figueiredo Frizzo, Carolina Araújo Rodrigues Funayama, Myriam Lima Isaac, José Fernando Colafêmina

**Affiliations:** 1 Master’s degree in neuroscience and doctoral student in neuroscience, Ribeirao Preto Medical School - USP. Professor at the Uberaba University, Graduate professor at the Ribeirao Preto University and the FIR/PE; 2 Lecturer, assistant professordoctor in the Neurology Department of the Ribeirao Preto Medical School - USP; 3 Assistant professor, Medical doctor of the Ophthalmology, Otorhinolaryngology, and Head & Neck Surgery Department in the Ribeirao Preto Medical School - USP; 4 Assistant professor, Medical doctor of the Ophthalmology, Otorhinolaryngology, and Head & Neck Surgery Department in the Ribeirao Preto Medical School - USP. Department of Neurology and Otorhinolaryngology - Ribeirao Preto Medical School - USP

**Keywords:** auditory evoked potentials, auditory evoked response

## Abstract

A**im**: To examine the components of auditory middle latency responses (AMLRs) in a sample of healthy children to establish their properties. **Methods:** Thirty-two children of both genders aged between 10 to 13 years, with no neurological disorders, were included in the study. Data were analyzed statistically by descriptive statistics (mean + SD) and by analysis of variance using the F test. AMLRs were investigated with toneburst stimuli at 50, 60 and 70 dB HL. **Results and Conclusions:** The mean latencies of the components were Na = 20.79 ms, Pa = 35.34 ms, Nb = 43.27 ms, and Pb = 53.36 ms, in 70 dB HL. The mean values for the NaPa amplitude ranged from 0.2 to 1.9 mV (M = 1.0 mV). The amplitude increased and latency decreased with increasing sound intensity. Inclination of the NaPa wave complex was present in some cases, which deserves attention in similar studies or in children with speech, language and auditory processing difficulties. **Conclusion:** This study provides additional information about AMLRs and may be a reference for others clinical and experimental studies in children.

## INTRODUCTION

Auditory middle-latency responses (AMLRs) consist of a wave sequence used for investigating the state of central auditory pathways.

Audiologists have given their approval to this diagnostic tool. Current medical application of this method, however, requires further studies to establish guidelines for wave identification in children. Maturity should be taken into account when this method it used for assessing central audition, meaning that great care is needed when interpreting AMLRs in children. Few papers on AMLRs during childhood have been published in the Brazilian medical literature. Although studies have been undertaken to investigate findings in pathological conditions, normal values for this procedure still need to be established. Wave shape abnormalities, especially in Pa and Na, may be seen in children with auditory processing disorders or auditory injury.

The aim of this paper is to describe AMLR findings at sound intensity levels of 50, 60, and 70 dB NA with tone burst stimuli in healthy children aged between 10 and 13 years.

### Auditory middle-latency responses (AMLRS)

AMLRs are the auditory responses evoked by the presentation of a sound stimulus; the responses occur 10 to 80ms after stimulation.^1^, ^2^ These rapid responses are measured in milliseconds (ms). Wave forms appear as positive (P) and negative (N) voltage peaks in sequence, represented alphabetically by lowercase letters that include the components Po, Na, Pa, Nb, Pb, and Nc. This form of representation was introduced by Goldstein and Rodman in 1967^3^ and has since been used universally. The Po wave is currently not considered a component of AMLRs, as it reflects mostly the electrical activity of the postauricular muscles.^4^ AMLRs are polyphasic waves that are highly consistent in the waking state and during sleep. Na, Pa, Nb and Pb are the most frequently analyzed waves due to their increased amplitude and consistency.5,7 The waveforms Na-Pa are also frequently used and investigated.^8^ The Pb wave is highly variable and may not appear in normal subjects.^4^

As in auditory brainstem responses (ABRs), the consistency of latency and amplitude facilitate wave identification. Pa is usually more robust, and may in this sense be compared to the V wave in ABRs.^2^, ^4^ If wave identification is done according to the consistency of AMLR latency and amplitude values, it becomes possible to avoid subjective interferences in marking the waves, increasing the reliability of the method.

Clinically, the study of AMLRs is a useful diagnostic tool for investigating the function of auditory pathways and for estimating auditory sensitivity.^9^ It is also helpful in studying central auditory function in patients with language, speech and learning disabilities with auditory processing disorders.^5^, ^10^, ^11^ Past studies have pointed to the simultaneous participation of multiple neural generators in eliciting the cortical electrical response. The inferior colliculus, the medial geniculate body, the reticular formation and the primary auditory area participate in generating AMLRs together with other associated areas and the corpus callosum. The reticular formation appears to be significantly related to primary and non-primary auditory pathways.^5^

Multiple neural generators form two systems of neural generators involved in generating AMLRs. One of them is the subcortical portion of the auditory pathway, which develops early; the other is the cortical portion that develops later. Development of a primary neural generator varies among individuals, but is complete at around ages 10 to 12 years. Pb is probably a cortical response with non-primary characteristics that originates in association areas; it is, therefore, not fully developed until adulthood.^12^ The result is that auditory pathway maturity interferes directly on the generation of AMLRs.

In younger children the presence of waves depends mostly of the state of sleep, where the generation of responses is predominantly subcortical (reticular formation).9 Hall4 stated that there is a complex interaction between latency and amplitude and the research subject’s age. The Pa wave amplitude is on average 1.0 µV in normal subjects. Amplitude is decreased and latency is increased in children below 10 years of age.

Based on this background the aim of this study was to investigate the components of AMLRs in a sample of healthy children for establishing the properties of AMLRs in this age group.

## MATERIAL AND METHODS

This study was approved by the Research Ethics Committee of the University Hospital of the Ribeirao Preto Medical School, USP (process number 3863/2001). Parents and caretakers signed a free informed consent form. The investigation was a cross-sectional historical cohort study involving 32 subjects (18 boys and 14 girls) aged between 10 and 13 years (mean: 11.7 years). Inclusion criteria were full term birth, absence of neurological disease, normal peripheral auditory system and registration in public schools with no requirements for special needs.

Subject selection and data collection included the clinical family and social history obtained from the files of the Centro Social Comunitário em Saúde da Vila Lobato (Vila Lobato Health Community Social Center), an institution registered in the Sao Paulo University in Ribeirao Preto, to review the subject’s pediatric history from birth to age 18 years.

Audiological data collection was done in the Auditory Perception Laboratory, Department of Psychobiology, College of Philosophy, Science and the Humanities, Sao Paulo University, in Ribeirao Preto. Patients underwent otoscopy, medical history-taking, and audiological assessments using a Maico MA-41 audiometer and a Teledyne Avionics TA3P (Washington, PA, EUA) middle ear analyzer. Pure tone air thresholds did not exceed 25 dB NA at 250 to 8000Hz13 and immitance testing revealed type A tympanometric curves.^14^

AMLRs were investigated with the ATI Nautilus PE, version 4.19 c software (Lermed S.R.L., Buenos Aires, Argentina) coupled to a single input channel preamplifier, an output amplifier, a computer and a TDH-39 earphone.

Subjects were accommodated in a reclining seat and the test room was protected from external acoustic and electrical interference. Subjects were instructed to keep their eyes open and to remain awake. The skin where electrodes were to be placed was cleaned with an abrasive paste to improve electrical conductivity; an electrolytic paste was used between the skin and the electrodes, which were attached with a microporous adhesive. The impedance of each electrode did not exceed 5 k ohms and the impedance difference between each electrode did not exceed 2 kΩ.^4^ Active electrodes were placed on the vertex (Cz) in reference to the right ear lobule (A2) and the left ear lobule (A1); alternate acquisition was used and the ground electrode was attached to the contralateral ear (International Federation 10-20 system15).

The method used 500 ipsilateral tone burst presented monaurally (plateau time = 6ms; rise/fall time = 2ms) at intensities of 50, 60 and 70 dB NA applied randomly at 1000Hz, using a 5 stimuli/second stimulation rate, a 70 or 100ms analysis time, a 3 to 100 Hz band-pass filter, alternate polarity, and sensitivity of 75µV. The mean testing time was about 35 minutes. Recordings were reproduced to assure reliability and waves were marked in tracings for easier replication.

Waves were identified based on the consistency of wave component latency and amplitude values. The wave component sequence and the replication of tracings resulted in the following: Na was the first highest negative peak between 12 and 27ms, Pa was the highest positive peak after the Na wave and between 25 and 40ms. Wave Pa was the most prominent of AMLR waves. Nb was the positive peak between 30 and 50ms immediately after Pa. Pb was the next highest positive peak between 45 and 65ms immediately following Nb.^1^, ^2^, ^3^, ^4^, ^8^

Identification of the Na-Pa-Nb complex facilitated wave visualization.^16^ Na-Pa amplitude values between peaks were also analyzed; these were marked from the first highest negative peak to the first highest positive peak between 0.5 - 2 µV and 0.4 - 2.58 µV, according to the literature.^7^, ^17^

AMLR amplitude analysis was used only for intraindividual comparison of values for each ear (Cz-A1/A2), reflecting neurophysiological baseline distinctions; this was the ear effect, which is due to limitations of the equipment. Each response in one side or the other could not be 50% lower in the same subject.^11^, ^18^ Normal levels were established using 2.5 standard deviations above or below the study population mean; this resulted in a 99.4% confidence level, which is similar to North-American standards for electrophysiological measurements.^19^

The SAS software was used for statistical analysis of the data. This included descriptive statistics (mean and standard deviation) and analysis of variance based on the F test, which took into account the effect of three factors, as follows: ear (A1 and A2), intensity (50, 60 and 70 dB) and waves (Na, Pa, Nb and Pb). The significance level was 5% in all of the analyses, and significant values are marked with an asterisk in tables.

## RESULTS

**Figure 1:**
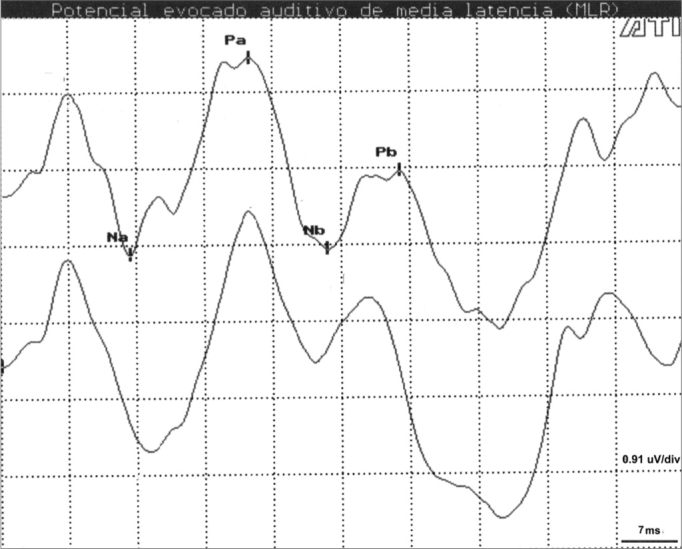
AMLR recording at 70 dBNA with replication. Key: mV= microvolts; ms = milliseconds.

Table 1 presents the results of descriptive statistics including wave latency means and the standard deviation (SD) for different intensities and electrode placements in Cz-A1/A2. Na, Pa and Nb components were observed in all subjects (100%) at high and middle intensities (Figure 1). Na-Pa waveforms were the most consistent and most easily identifiable components, showing high reproducibility and a 100% detectability rate. The Pb component was not identified in only 4 (2.0%) of 192 measurements.

**Table 1:** Mean (M) and Standard Deviation (SD) of wave latencies at the different intensity levels for Cz-A1/A2 (n = 32).

	Cz-A2 Latency (ms)								Cz-A1 Latency (ms)							
Intensity dB LH)	Na		Pa		Nb		Pb		Na		Pa		Nb		Pb	
	M	SD	M	SD	M	SD	M	SD	M	SD	M	SD	M	SD	M	SD
50	22.3	3.5	34.1	4.3	43.2	5.2	55.1	5.5	22.7	3.2	36.5	4.0	44.9	5.0	56.8	4.6
60	22.4	2.5	34.7	3.9	43.0	5.4	54.9	5.0	22.5	2.1	35.4	3.7	43.8	4.2	54.9	4.8
70	20.6	2.9	34.8	4.5	42.7	5.8	55.6	5.9	20.9	2.8	35.8	5.4	43.8	6.5	55.1	6.3

There were 4 measurements in the study population where latency values exceeded 2.5 SDs. 0.51% of measurements in 4 subjects were out of standard although the 98.99% confidence interval was maintained. In the interaural comparison that sought the ear effect only 6 subjects where shown to had amplitudes below 50%.

Reproducibility of tracings was partial (65.1%), and intra and intersubject variability was considerable, regardless of the intensity that was being investigated. However, lower SD values were obtained for the Na wave (Table 1). Na-Pa amplitude values varied from 0.15 to 1.9 µV (M = 1.0 µV) (Table 2).

**Table 2:** Mean (M) and Standard Deviation (SD) of wave amplitudes in the different intensity levels for Cz-A1/A2 (n = 32).

Intensity (dB HL)	Cz-A2 Amplitude (µV) Na-Pa			Cz-A1 Amplitude (µV) Na-Pa		
	M	Min	Max	M	Min	Max
50	0.81	0.16	1.60	0.53	0.15	1.70
60	0.99	0.20	1.90	0.82	0.28	1.50
70	1.05	0.16	1.90	1.02	0.38	1.90

Table 3 shows the mean and the variance of measurements at different intensities for the Cz-A1/A2 electrode derivation. There was no significant difference in most measurements, but at an intensity of 50 dB NA, Cz-A1 values were prolonged compared to Cz-A2.

**Table 3:** Latencies mean values and variances in the Ear Effect analysis.

Intensity (dB HL)	Cz-A2	Cz-A1	Test F
			(p ≤ 0.05)
50	38.76	40.27	8.70** (p = 0,003)
60	38.82	39.21	0.58
70	38.43	38.95	1.01

Test F	0.32	3.75* (p = 0.02)	

Table 4 presents the means for the Na wave at an intensity of 50 dB NA, which was significantly higher than the mean obtained at an intensity of 70 dB NA. There was, however, no significant difference between measurements obtained for the Na wave at an intensity of 60 dB NA. No significant difference was seen in Pa, Nb and Pb waves at intensities of 50, 60 and 70 dB NA.

**Table 4:** Latencies mean values and variances for different intensity levels and waves.

Intensity (dB HL)	Na	Pa	Nb	Pb
50	22.56	35.35	44.09	56.07
60	22.49	35.13	43.44	55.01
70	20.79	35.34	43.27	55.36

F Test	3.83* (p = 0.02)	0.76	0.72	1.11

**Figure 2:**
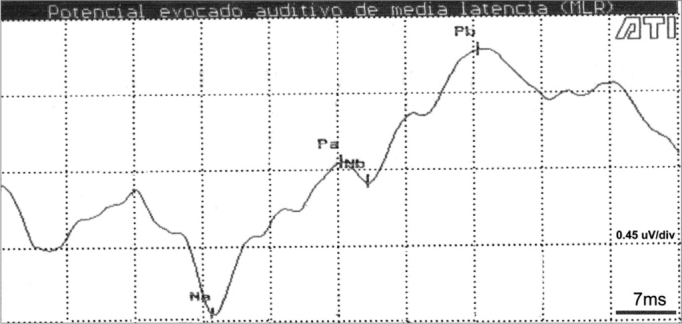
AMLR recording at 70dBNA in a 13-year-old child Key: mV= microvolts; ms = milliseconds.

In a few cases (4 children) the Na-Pa complex was inclined, associated with a prolonged Na wave latency, a reduced Pa wave amplitude and a negative Nb wave. This made identification of the Pa and Nb waves difficult (Figure 2).

## DISCUSSION

Na, Nb and Pa were the AMLR components with the highest identification rate (100%) at all the intensities we investigated. According to Ozdamar and Kraus,5 Na and Pa were also found in 100% of cases and Nb was identified in about 85% of cases. Pb was absent in 2% of our measurements irrespective of intensity. The Pb wave detectability rate varies from 30 to 50% in the literature.^3^, ^5^ Pb is a cortical component, especially from the associative or non-primary auditory cortex.12,20 It appears, therefore, that there is less interference from the exogenous response (frequency of the stimulus) when this wave is generated.

Na, Pa, Nb and Pb wave latencies were slightly prolonged in our study compared to results presented in other papers that investigated children of similar age.^11^, ^16^ Longer latencies are obtained using tones, compared to clicks.^5^, ^21^

The significant variability of latency values may be related to Ozdamar and Kraus’s5 observation of a high variation level of AMLR components compared to the ABR. On the other hand, Goldstein and Rodman3 noted that AMLRs had a consistent response pattern for low, middle and high intensities. Mendel and Goldstein6 stated that latency of components is related to their variability, where longer latencies are associated with higher wave variations. We obtained lower SDs for the Na wave, and only 4 measurements exceeded 2.5 SDs, resulting in a confidence level of 98.99%. This method, therefore, may be used with a favorable confidence level, and suggests statistical homogeneity of data.

The consistency of Na-Pa waveforms and the Na-Pa amplitude seen in this study was in accordance with the literature.^11^, ^18^ On the other hand, myogenic responses that occur at similar latencies may interfere with recordings and forestall full reproducibility of the tracings.^4^ This may have contributed to the partial reproducibility rate (65.1%) of our neural recordings.

The inclination of the Na-Pa complex, prolongation of the Na wave latency, the reduction of the Pa wave amplitude and the Nb wave negativity, all of which were present in some cases, configure a possible morphology according to Ozdamar and Kraus.^5^ However, it is worth noting that during clinical history-taking, children with this recording pattern complained of difficulties at school, which suggests that healthy children should also be investigated as well as those in pathological conditions.

According to Kraus et al.^16^ and Hall^4^, wave latencies are equivalent in ipsilateral and contralateral modes of stimulation. Other investigators^22^, however, have reported that latency is lower in contralateral recordings compared to ipsilateral recordings; these authors also agree that ipsilateral stimulation prolongs wave latency.

The relation between increased intensity and reduced latency in our study confirms previous observations^3^, ^4^ that show progressive increases at intensities of up to 40-50 dB SPL over the tone threshold of subjects and a gradual decrease of the wave latency. At higher intensities, wave latency remains relatively constant. According to Borgmann et al., ^21^ if a tone stimulus is used, a 40-80 dB NA intensity increase produces a small decrease in final wave latency; this effect is mostly observed in initial waves.

McGee and Kraus12 have reported the AMLR change characteristics not only in wave morphology and neurodevelopmental terms, but also in terms of response safety. These authors contend that cortical and subcortical neural components are responsible for generating the Pa wave. In younger children these responses are attributed mainly to subcortical neural generators that develop earlier. The response in adults is dominated by cortical neural generators that develop later. A disorder involving the AMLR generating system may delay development or result in AMLR abnormalities. These changes may be best defined within the context of neural development and maturation together with precise information about normal characteristics in children, which is the aim of this paper.

## CONCLUSION

This paper adds information about AMLRs and may be useful as a reference for further clinical or experimental studies in healthy children or in children with speech, language and auditory processing difficulties.
